# Quantifying the Impact of Woodpecker Predation on Population Dynamics of the Emerald Ash Borer (*Agrilus planipennis*)

**DOI:** 10.1371/journal.pone.0083491

**Published:** 2013-12-09

**Authors:** David E. Jennings, Juli R. Gould, John D. Vandenberg, Jian J. Duan, Paula M. Shrewsbury

**Affiliations:** 1 Department of Entomology, University of Maryland, College Park, Maryland, United States of America; 2 Animal and Plant Health Inspection Service, United States Department of Agriculture, Center for Plant Health Science and Technology, Buzzards Bay, Massachusetts, United States of America; 3 Agricultural Research Service, United States Department of Agriculture, Robert W. Holley Center for Agriculture and Health, Ithaca, New York, United States of America; 4 Agricultural Research Service, United States Department of Agriculture, Beneficial Insects Introduction Research Unit, Newark, Delaware, United States of America; University of Western Ontario, Canada

## Abstract

The emerald ash borer (EAB), *Agrilus planipennis*, is an invasive beetle that has killed millions of ash trees (*Fraxinus* spp.) since it was accidentally introduced to North America in the 1990s. Understanding how predators such as woodpeckers (Picidae) affect the population dynamics of EAB should enable us to more effectively manage the spread of this beetle, and toward this end we combined two experimental approaches to elucidate the relative importance of woodpecker predation on EAB populations. First, we examined wild populations of EAB in ash trees in New York, with each tree having a section screened to exclude woodpeckers. Second, we established experimental cohorts of EAB in ash trees in Maryland, and the cohorts on half of these trees were caged to exclude woodpeckers. The following spring these trees were debarked and the fates of the EAB larvae were determined. We found that trees from which woodpeckers were excluded consistently had significantly lower levels of predation, and that woodpecker predation comprised a greater source of mortality at sites with a more established wild infestation of EAB. Additionally, there was a considerable difference between New York and Maryland in the effect that woodpecker predation had on EAB population growth, suggesting that predation alone may not be a substantial factor in controlling EAB. In our experimental cohorts we also observed that trees from which woodpeckers were excluded had a significantly higher level of parasitism. The lower level of parasitism on EAB larvae found when exposed to woodpeckers has implications for EAB biological control, suggesting that it might be prudent to exclude woodpeckers from trees when attempting to establish parasitoid populations. Future studies may include utilizing EAB larval cohorts with a range of densities to explore the functional response of woodpeckers.

## Introduction

Since it was accidentally introduced to North America in the 1990s the emerald ash borer (EAB), *Agrilus planipennis*, has killed millions of ash trees (*Fraxinus* spp.), and the estimated costs for future management are over $10 billion in the next decade [1]–[3]. Differential host resistance is thought to be primarily responsible for the successful establishment and spread of EAB in North America [4]–[6], but the extent to which this is influenced by a lack of natural enemies in their introduced range remains unclear. For example, in Asia, major sources of mortality for EAB larvae include a complex of parasitoids and predators as well as tree resistance [7], [8]. Consequently, determining the relative importance of these mortality factors in EAB's introduced range should help guide efforts to manage it.

In addition to parasitism and tree resistance, predation from insectivorous birds such as woodpeckers (Picidae) can be a substantial source of mortality for EAB larvae in their native range [7], [8]. Woodpecker predation has been found to account for approximately 25% of EAB mortality in China [7], while in Russia it may reach approximately 40% [8]. Indeed, even in North America, woodpeckers appear to have quickly responded to EAB populations, often causing mortality rates between 35–40% [9]–[12] and with reports of up to 95% EAB mortality in individual trees [13]. To date these studies have been conducted in Michigan (the epicenter of the EAB invasion in North America, where it was first detected in 2002), and consequently woodpeckers may have had sufficient time to both adapt their foraging behavior and respond numerically to this novel prey species. While these studies have provided important data on woodpecker predation in areas heavily infested by EAB, examining predation in the newly invaded region or at the edge of the current range should also provide useful data for parameterizing models predicting future rates of EAB population growth. Furthermore, to the best of our knowledge no previous studies have experimentally manipulated woodpecker predation to tease apart its relative contribution to EAB larval mortality.

Life tables have widely been utilized in ecology and entomology to examine how mortality and fecundity affect population dynamics in myriad species [14]–[16], and they can be particularly useful when implementing biological control for invasive species [17]–[19]. Because of their relatively long life cycle and cryptic immature stages, construction of a complete life table for EAB poses distinct challenges. For example, after hatching from eggs laid in bark crevices EAB larvae proceed to feed in the cambium of host trees where they will go through four larval instars over summer and fall (though in colder climates and/or healthier trees this stage can last into a second summer). EAB then fold into a J-shape and overwinter as “J-larvae” [20], also termed “prepupae” by Chamorro et al. [21]. Development is completed the following spring with EAB emerging as adults in early summer [22]. However, Duan et al. [20] showed that by experimentally creating cohorts of EAB larvae it is possible to identify the causes of mortality that occur during their development under the bark of trees.

In the present study we utilized two approaches to quantify woodpecker predation on EAB in natural stands of ash. First, we sampled wild populations of EAB larvae in ash trees at heavily infested sites in New York, where screening was used to exclude woodpeckers from sections of each individual tree. Second, we established experimental cohorts of EAB larvae in ash trees at sites with low levels of EAB infestation in Maryland, where cages were constructed around half of the study trees to exclude woodpeckers. We have anecdotally observed several species of woodpecker foraging on ash trees in both locations, including the downy woodpecker (*Picoides pubescens*) in New York, and the red-bellied (*Melanerpes carolinus*) and hairy woodpeckers (*P*. *villosus*) in Maryland (C. Pickett, pers. comm.). Our specific objectives were: 1) to determine the relative contribution of woodpecker predation to overall EAB mortality and 2) to use data from the wild populations and experimental cohorts to construct life tables and model potential EAB population growth with and without woodpecker predation. Because some woodpeckers can prefer feeding on dead or dying trees [23], we predicted that woodpecker predation would be a greater source of mortality at sites with a more established EAB infestation. Additionally, we predicted that EAB population growth would be higher in the absence of woodpecker predation.

## Materials and Methods

### Ethics statement

We would like to thank Jesse Morgan and Mark Beals of Green Ridge State Forest, and Sal Culoso, Russell Cashdollar, Kathleen Sheehan, Andrew Muszynski, and Dorothy Gragg for permissions to use field sites.

### Study sites

In New York our study sites were located in a mature EAB infestation in Ulster County. EAB populations were discovered in 2010, however EAB most likely arrived around 2002 (N. Siegert, pers. comm.). We selected stands of white ash (*F*. *americana*) at five sites that had high densities of EAB. The sites were a minimum of 1.5 km apart. At each site we selected six ash trees that showed signs of EAB infestation (e.g., canopy reduction or epicormic growth) because at these locations we relied on natural oviposition to infest the trees.

In Maryland our study was conducted at 12 sites, all with low to medium levels of previous EAB infestation (D. Bean, pers. comm.). Six of the sites were located in eastern Allegany County (EAB first detected in 2011), and the other six were located around southwestern Prince George's County and northeastern Charles County (EAB first detected in 2010). Individual sites were located a minimum of 1.5 km apart, and introduced larval parasitoids (*Spathius agrili* and *Tetrastichus planipennisi*) had previously been released at eight of the sites. Belt transects (100 m long and 10 m wide) were used initially to determine the species, density, and condition of ash trees at each site. Within each transect we recorded the diameter at breast height (DBH) and crown condition of all ash trees present. Crown condition of trees was assessed using the method described by Smith [24], with class 1 crowns representing the healthiest trees and class 5 crowns representing trees with >80% reduction in crown cover. At each site we selected 10 green ash trees (*F*. *pennsylvanica*) (n = 120, mean DBH = 13.39 cm, SE±0.35) that appeared healthy and un-infested by EAB on which to establish our experimental cohort (n = 120, mean crown condition = 2.08, SE±0.09). We attempted to use trees that appeared un-infested by EAB as high levels of infestation can result in galleries from wild EAB larvae obscuring those from the experimental cohort [20].

### Wild population sampling

For our sampling of wild EAB populations in New York we selected six trees per site (n = 30, mean DBH = 21.54 cm, SE±0.98) that showed symptoms of EAB infestation. On the lower 2.5 m of the tree we marked off two 1 m sections of trunk beginning 0.5 m above the ground. On half of the trees the lower section was covered in standard metal window screening to exclude woodpeckers, and the upper section was screened on the remaining trees (for a total of 60 tree sections). The screening was placed flush against the trunk of the tree and stapled in place in August 2011. We measured the diameter of the tree at the mid-point of each 1 m section. Although some woodpeckers might commonly forage high up in trees [25], our anecdotal observations and other previous research [20], [23] have indicated that they would search for prey within the heights of the tree sections examined in this study.

### Experimental cohort creation

Using a modified version of the methods of Duan et al. [20], in the summer of 2012 we experimentally established cohorts of 30 EAB eggs on the lower 2.5 m of each of the 10 trees selected at each site in Maryland. The eggs used were laid on filter paper by laboratory-reared females before the filter paper was cut into thin strips each hosting one to three eggs. Eggs were then attached to the tree in five bands (approximately 30 cm apart to minimize the chance of larval galleries overlapping), with each band consisting of six eggs. To attach eggs to the tree, we first scraped a patch of bark flat using a draw knife. A strip of filter paper was then glued flush to the bark (using standard wood glue) before being covered by a cotton ball (to minimize predation by ants). After each row of eggs had been attached, all cotton balls were covered with tree wrap (to reduce predation risk further). To exclude woodpeckers, the experimental cohorts on half of the trees at each site were covered with 20-gauge galvanized hex netting with 2.5 cm openings in August 2012. The netting was suspended above the cohorts from two nails and reached down to the base of the tree such that it was hanging approximately 5 cm away from the bark surface, preventing woodpeckers from reaching the trunk and ensuring all EAB larvae on the tree were caged.

### Determining the fate of larvae

In late winter 2012 (New York) and 2013 (Maryland) all of the trees containing wild populations or experimental cohorts were debarked up to a height of 2.5 m and the fates of EAB larvae were determined. Fates of larvae were assigned into one of six categories: 1) development to adulthood (indicated by a D-shaped exit hole from a pupal chamber), 2) live larva, 3) killed by tree resistance (indicated by the tree forming a callous around the gallery), 4) parasitized (indicated by the presence of parasitoid larvae, adults, cocoons, pupae or meconium in the gallery), 5) preyed upon (indicated by part or all of the larva missing, in addition to bark damage from woodpeckers), or 6) diseased (indicated by a decomposing cadaver) [20]. We were able to locate where larvae of the experimental cohorts entered the tree and started creating their gallery by their proximity to where eggs were attached, and thus we were able to distinguish them from wild larvae. This enabled us to identify any wild EAB larvae present, and these numbers were used to provide an estimate of wild EAB density per tree (based on the number of wild EAB larvae found per m^2^ of tree surface area debarked).

### Data analyses

**Figure 1:**
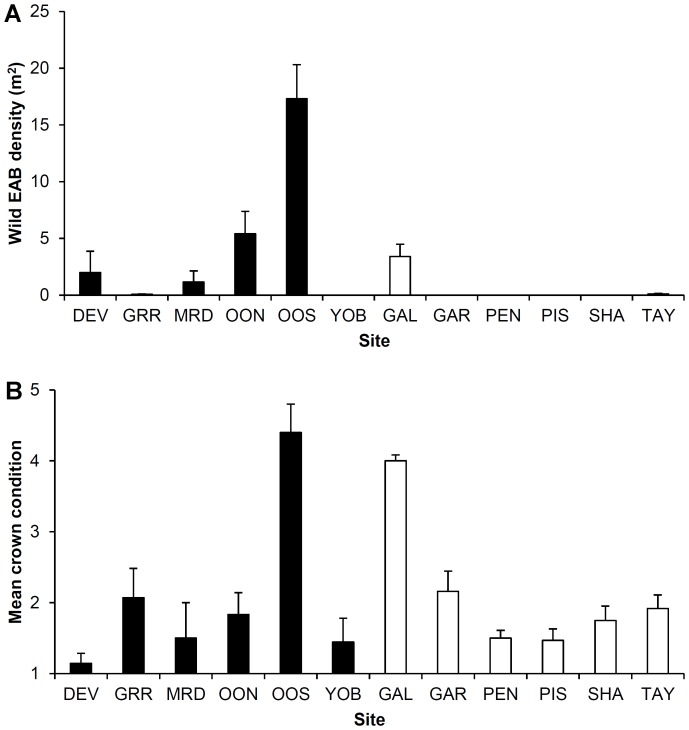
Mean wild EAB density (A) and site crown condition (B) at sites in Maryland. Crown condition was measured on a scale of 1–5, with 1 representing healthy trees and 5 representing trees with >80% reduction in crown cover. Shown are sites located in eastern Allegany County (solid) and sites in southwestern Prince George's County and northeastern Charles County (open), with standard errors.

All statistical analyses were conducted using R 2.15.2 [26]. In the wild populations of EAB larvae in New York, we were interested in examining how the percentage of larvae found in each fate category (determined from the total number of larvae found in each tree section) varied by site and differed between open and screened sections of trees. Similarly, for our experimental cohorts in Maryland we wanted to ascertain how the percentage of larvae in each fate category (determined from the number that had successfully hatched from eggs on each tree) differed between open and caged trees, or varied with individual tree crown condition, density of wild EAB larvae, or mean crown condition of trees per site (based on data from the belt transects, and hereafter referred to as ‘site crown condition’). However, tree crown condition and wild EAB density were highly correlated (Spearman's rho = 0.720, *P*<0.001), so tree crown condition was dropped from the models. When analyzing the experimental cohorts we included wild EAB density (Figure 1A) and site crown condition (Figure 1B) in models to account for site-specific differences in previous EAB infestation.

Data on larval fates from both wild populations and experimental cohorts were first tested for normality using the Shapiro-Wilk test using the ‘shapiro.test’ function in the ‘stats’ package [26]. None of the data on larval fates met the assumptions of normality (all *P*<0.05), and therefore they were fit to generalized linear models with a binomial error distribution and logit link using the ‘glm’ function in the ‘MASS’ package [27]. The significance of models was then assessed using chi-square tests based on log-likelihood ratios using the ‘Anova’ function in the ‘car’ package [28], and models were tested for all main effects.

To construct life tables we followed the general methods and column definitions described in Southwood and Henderson [29]: nx – the number of live EAB entering each stage; dx – the number of EAB dying in each stage; lx – the proportion of EAB surviving each life stage; qx – the mortality rate for each life stage; mx – the age-specific fertility rate (female offspring only); and lxmx – the average number of female offspring per stage. Net reproductive rate (R_0_) was then calculated by summing the lxmx column. The number of live EAB entering each stage was estimated based on reverse calculation of the different stages of EAB observed at the sampling time. Because of the destructive nature of our sampling (and to avoid releasing EAB adults into the wild), we were unable to generate our own field data for adult fecundity or sex ratios. Therefore we used data on EAB previously obtained by Wang et al. [7] for these parameters in our life tables and assumed constant fecundity for all treatments.

## Results

### Wild populations

**Figure 2:**
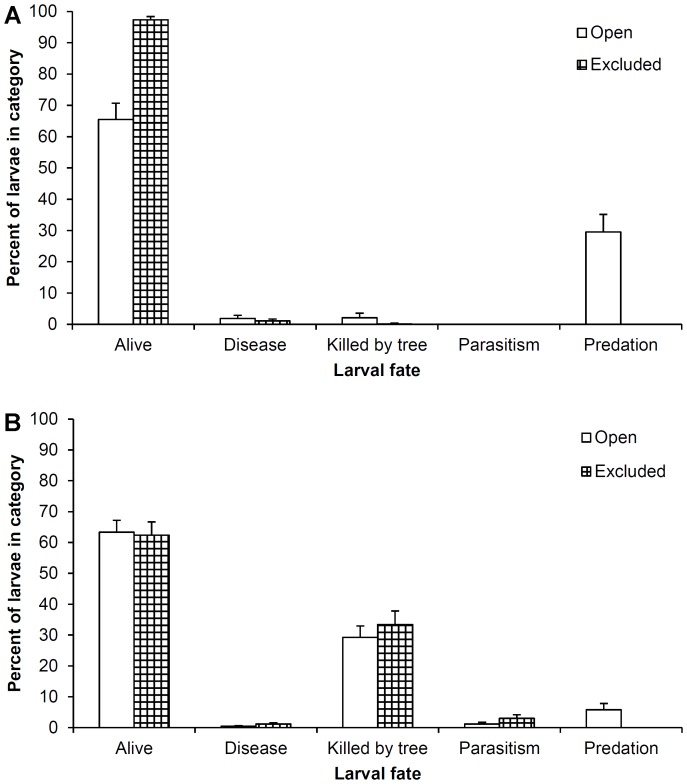
Overall fates of EAB larvae in wild populations (A) and experimentally established cohorts (B). Shown are trees from which woodpeckers were excluded (hashed) and trees left open and exposed to woodpecker predation (open), with standard errors.

One tree sampled for wild populations of EAB was found to be dead, reducing the number of individual trees included in the analyses to 29. We were able to determine the fates of 1,528 larvae from these 58 tree sections (the fates of a further 14 larvae were categorized as ‘un-ascribed’), with seven tree sections found to contain no larvae. The vast majority of larvae from the wild populations were categorized as being alive (1,176) at the time of sampling. Of these living EAB, most were found to be in the pupal stage (71.3%), followed by L4 (11.6%), L3 (7.2%), and L2 (6.1%) larval instars. Smaller numbers of EAB were found as prepupae (3.2%), adults that had emerged (0.3%), and J-larvae (0.3%). There was a significant difference in the percentage of larvae found alive between screening treatments (Table 1), with considerably more living larvae present when woodpeckers were excluded by screening (97.3%) compared to when they were exposed to predation from them (65.5%) (Figure 2A). The position of the screening on the tree was also significant, with more larvae typically found alive in the lower sections of trees (85.8%) compared with the upper sections (75.3%) (Table 1). Additionally, we found significant variation in the percentage of larvae found alive among sites (range 75.7% to 84.1%).

**Table 1:** Results from likelihood ratio tests for the effects of screen (presence or absence of screen excluding woodpeckers), position (whether the screen was on the lower or upper section of the tree), and site on the fates of EAB larvae from wild populations.

Factor	Larval fate			
	Alive	Diseased	Killed by tree	Predation
Screen	423.70 ***	0.02	15.36 ***	518.22 ***
Position	99.67 ***	0.28	2.81	132.79 ***
Site	16.86 **	8.39	22.73 ***	26.98 ***

Shown are χ^2^ values (* *P*<0.05, ** *P*<0.01, *** *P*<0.001).

A total of 324 larvae were found to have been depredated by woodpeckers, making it the second most common larval fate and the main source of mortality in the wild populations sampled. Woodpecker predation was found to occur almost exclusively on J-larvae, prepupae, and pupae (98.8%), with a remainder occurring on L4 larval instars (1.2%). Screening treatment had a significant effect on the percentage of larvae depredated by woodpeckers (Table 1), with no evidence of woodpecker predation when screens were present (0%) compared to 29.6% of larvae being depredated when exposed to woodpeckers (Figure 2A). Woodpecker predation on larvae was significantly higher in exposed upper sections (21.1%) of trees compared to exposed lower sections (9.9%) (Table 1), and we found a significant difference in the amount of woodpecker predation among sites (range 8.6% to 19.9%) (Table 1).

The remaining larval fates comprised an extremely small amount of the total larvae found. While we did detect a significant difference in the percentage of larvae killed by tree resistance between screening treatments (Table 1; Figure 2A), we found only 14 larvae in this category. A further 14 larvae were found to be diseased, and there was no evidence of any larvae being parasitized (Figure 2A).

**Figure 3:**
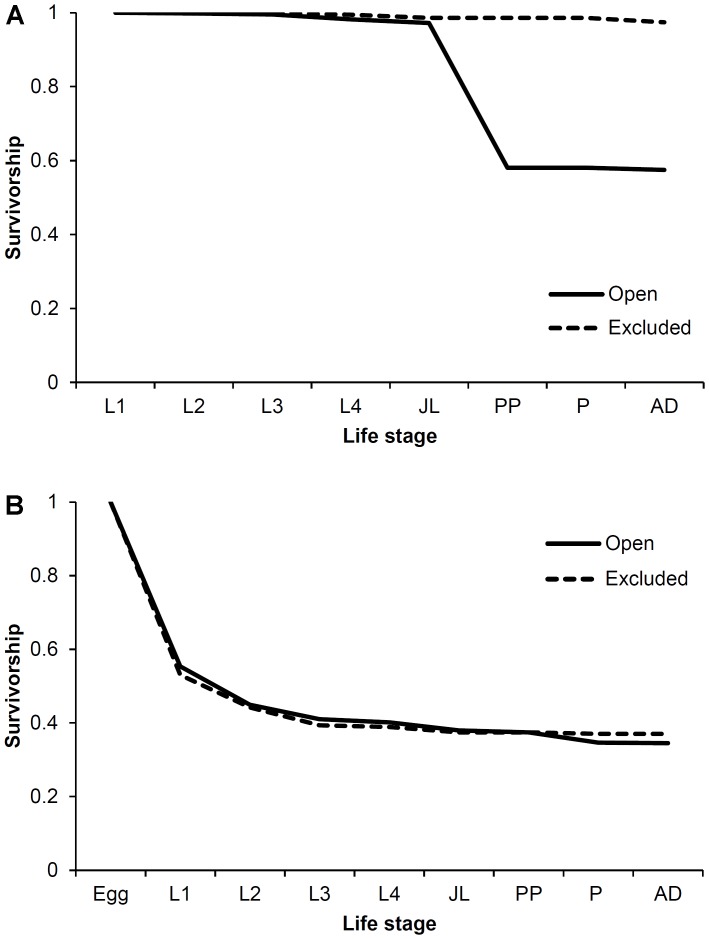
Stage-specific survivorship of EAB in wild populations (A) and experimentally established cohorts (B). Dashed line represents survivorship in trees from which woodpeckers were excluded and solid line represents survivorship in trees left open and exposed to woodpecker predation.

We were able to construct partial life tables and generate R_0_ for EAB populations in sections of trees that were exposed to woodpecker predation as well as those that were screened. Because larvae in these trees originated from natural oviposition, we were not able to calculate the hatching success rate at the egg stage. An R_0_>1 indicates a population that is growing, and clearly both EAB populations (open and screened) were growing as they exhibited high R_0_ (Table 2). There was a considerable difference between the R_0_ values in open and screened populations, with screened populations where woodpeckers were excluded having much higher survivorship and growth rates (Figure 3A). We assume that all larvae discovered represented one generation in these wild populations. It is possible some individuals were engaged in a two-year cycle. However, given the difficulties associated with establishing and identifying experimental cohorts of larvae in heavily infested trees, we think the effect of this did not significantly influence our results.

**Table 2:** Life table for wild populations of EAB in trees left open and exposed to woodpecker predation (O) and trees screened to exclude woodpeckers (E).

Life stage	nx		dx		lx		qx		mx		lxmx		R_0_	
	O	E	O	E	O	E	O	E	O	E	O	E	O	E
L1	818	724	2	0	1	1	0.003	0						
L2	816	724	2	1	0.998	1	0.003	0.001						
L3	814	723	11	3	0.995	0.999	0.014	0.004						
L4	803	720	8	6	0.982	0.995	0.010	0.008						
J-larvae	795	714	320	0	0.972	0.986	0.403	0						
Prepupae	475	714	0	0	0.581	0.986	0	0						
Pupae	475	714	5	9	0.581	0.986	0.011	0.013						
Adult	470	705	470	705	0.575	0.974	1	1	51.835	51.835	29.783	50.475	29.783	50.475

Column headings represent: nx – the number of live EAB entering each stage; dx – the number of EAB dying in each stage; lx – the proportion of EAB surviving each life stage; qx – the mortality rate for each life stage; mx – the age-specific fertility rate (female offspring only); lxmx – the average number of female offspring per stage; and R_0_ – net reproductive rate.

### Experimental cohort

We were unable to collect data from four study trees with previously undetected high levels of wild EAB infestations. These beetles interfered with the experimental cohorts and prevented us from determining the fates of larvae with any certainty. Of the remaining 3,480 eggs placed on 116 trees, 1,882 (54.1%) successfully hatched into larvae. We subsequently found 1,249 larvae that were still alive, making it the most common overall fate in our EAB cohorts. At the time of sampling most of the living larvae were in the prepupal stage (62.3%), followed by the pupal (13.6%), and J-larval (7.5%) stages. The remaining living larvae were observed to be in the L2 (6.2%), L3 (5.3%), L4 (4.2%), and L1 (0.9%) instars. Of these early instar larvae, 88% were found at the sites in Allegany County which suggests that perhaps a two-year life-cycle is more common there (possibly because of differences in tree health or climate). We did not find any larvae that had exited as adults by the time our survey was conducted, which was not surprising as EAB adults typically emerge in early May in Maryland.

For the cage experiment, the percentage of larvae found alive did not vary significantly between open (63.4%) and caged (62.4%) trees (Figure 2B) (Table 3). The percentage of living larvae also did not vary significantly with site crown condition (Table 3). However, the percentage of larvae found alive did vary significantly with wild EAB density (Table 3).

**Table 3:** Results from likelihood ratio tests for the effects of cage (presence or absence of cage excluding woodpeckers), site crown condition, and wild EAB density on the fates of EAB larvae from experimentally established cohorts.

Factor	Larval fate				
	Alive	Diseased	Killed by tree	Parasitism	Predation
Cage	0.21	6.41 *	0.78	10.12 **	96.30 ***
Site crown	3.28	1.08	20.23 ***	58.33 ***	82.72 ***
Density	62.56 ***	0	110.75 ***	0.25	7.15 **

Shown are χ^2^ values (* *P*<0.05, ** *P*<0.01, *** *P*<0.001).

The second most common fate (and the largest source of mortality) for EAB larvae in our experimental cohorts was the killed by tree category, which accounted for a total of 500 larvae. There was little difference in the percentage of larvae that were killed by tree resistance between open (29.3%) and caged (33.4%) trees, and it was not significant (Table 3; Figure 2B). However, there were significant differences between the percentage of larvae killed by trees and site crown condition and wild EAB density (Table 3).

We found evidence of woodpecker predation on a total of 64 larvae in 10 trees from three sites. Larvae in trees exposed to woodpeckers experienced a higher level of predation (5.8%) than those in caged trees (0%), and this difference was significant (Table 3; Figure 2B). Moreover, on the 10 trees that received any level of woodpecker predation, it accounted for an average of 76.3% (range 42.1% to 100%) of the mortality for larvae on that tree. Site crown condition and wild EAB density were also significant predictors of woodpecker predation (Table 3).

Parasitism was observed on a total of 51 larvae in 18 trees at eight sites, and the parasitoids most commonly encountered were the introduced *T*. *planipennisi* (31.4%) and *S*. *agrili* (17.7%), and the native *Balcha indica* (19.6%) and *Atanycolus* sp. (15.7%). The percentage of larvae parasitized differed significantly between open (1.2%) and caged (3%) trees (Figure 2B), and site crown condition (Table 3). There was no significant effect of wild EAB density (Table 3).

Larvae that were diseased comprised the smallest fate category, with only 18 total occurrences in 12 trees at seven sites. Although relatively few larvae were found in this category, we did detect a significant difference in the percentage of larvae that were found diseased between open (0.4%) and caged (1.2%) trees (Figure 2b), but not between site crown condition or wild EAB density (Table 3).

Life table analysis for the experimentally established EAB cohorts indicated that both (open and caged) populations were growing as they possessed high R_0_ (Table 4). Although EAB larvae in trees from which woodpeckers had been excluded had slightly higher survivorship and R_0_ than trees exposed to woodpecker predation (Figure 3B), the difference was far smaller than what we found in the wild populations. Additionally, we observed that most woodpecker predation on EAB occurred in J-larval, prepupal or pupal stages (71.9%), with the remainder occurring in the L4 larval instar (28.1%).

**Table 4:** Life table for experimentally established EAB cohorts in trees left open and exposed to woodpecker predation (O) and trees caged to exclude woodpeckers (E).

Life stage	nx		dx		lx		qx		mx		lxmx		R_0_	
	O	E	O	E	O	E	O	E	O	E	O	E	O	E
Egg	1590	1890	710	888	1	1	0.447	0.470						
L1	880	1002	166	167	0.554	0.530	0.189	0.167						
L2	714	835	62	91	0.449	0.442	0.087	0.109						
L3	652	744	13	9	0.410	0.394	0.020	0.012						
L4	639	735	35	27	0.401	0.389	0.055	0.037						
J-larvae	604	708	9	0	0.380	0.375	0.015	0						
Prepupae	595	708	44	8	0.374	0.375	0.074	0.011						
Pupae	551	700	2	0	0.347	0.370	0.004	0						
Adult	549	700	549	700	0.345	0.370	1	1	51.835	51.835	17.898	19.198	17.898	19.198

Column headings represent: nx – the number of live EAB entering each stage; dx – the number of EAB dying in each stage; lx – the proportion of EAB surviving each life stage; qx – the mortality rate for each life stage; mx – the age-specific fertility rate (female offspring only); lxmx – the average number of female offspring per stage; and R_0_ – net reproductive rate.

## Discussion

Our results indicate that woodpecker predation can comprise an important source of mortality for EAB. However, comparing trends from the wild populations and experimentally established cohorts it is apparent that woodpecker predation is highly dependent on factors such as the maturity of the EAB infestation and the associated site crown condition and wild EAB density. These findings are consistent with our predictions and similar to those of Lindell et al. [9], who also observed that within sites woodpeckers tended to forage on trees in poorer condition and with higher wild EAB densities. While woodpeckers frequently have been shown to respond positively to pest insects including EAB [30]–[33], their ability to protect ash could be limited because of this preference for foraging on trees in poor condition that are already heavily infested. Dead and dying trees often also provide nesting sites for woodpeckers [34]–[36], and fewer nesting sites available in the initial stages of EAB invasion could delay the numerical response of woodpeckers [37].

There was a considerable difference between New York and Maryland in the effect of woodpecker predation on predicted EAB population growth, lending further support to the idea that woodpecker predation is likely to be dependent on the time since EAB colonization (because of the time lag in the response of woodpeckers to changes in EAB population size and habitat quality). EAB invasions have been described as having three main stages: the cusp, crest, and core [38]. The cusp phase occurs at newly infested sites over the first few years as EAB populations slowly build, before their numbers rapidly increase and cause high tree mortality in the crest phase. The core phase then occurs around 10 years after the initial infestation by which time most trees have died. Our studies were conducted at sites in the crest (New York) and cusp (Maryland) phases, which likely explains the variation in the level of woodpecker predation we observed. Specifically, while the level of woodpecker predation we found in New York was relatively similar to those found in other North American locations such as Michigan [9]–[11], [13], [20], the level we recorded in Maryland were generally much lower than these. Alternatively, our results may simply indicate that woodpecker populations were already more established at our sites in New York compared with those in Maryland.

The significant difference in parasitism that we detected between open and caged treatments in Maryland could have implications for EAB biological control. At present two larval parasitoids have been released to suppress EAB, *T*. *planipennisi* and *S. agrili* [39], [40], and there also are several native larval parasitoids that have become associated with EAB [11], [41], [42]. The introduced parasitoids have had some success in establishing at release sites [12], though presently their effect on EAB populations is unclear. Our results suggest that it could be prudent to deploy screening or caging to exclude woodpeckers from certain heavily infested trees at new release sites in order to expedite the establishment of parasitoids. We do not hypothesize that woodpeckers would prey differentially on parasitized larvae [12], but any predation on these larvae might interfere with the parasitoid population growth. Moreover, our observation of increased parasitism when there are more larvae (i.e., when they are protected from woodpeckers) could provide support for the compensatory mortality hypothesis [43], and indicate that the parasitoids are exhibiting density-dependent attack behavior. However, it is difficult to identify density-dependent attack behavior from the present study because EAB parasitoids generally attack larvae in the summer, before they have reached a size sufficiently large to interest woodpeckers.

In terms of the remaining sources of EAB larval mortality, our results are largely consistent with previous findings [11], [12], [20]. For example, it was not surprising to observe that the general condition of trees at each site, and the condition of individual trees within a site both have a large effect on the fate of larvae. Larvae that were categorized as being killed by tree resistance in our experimental cohorts were often in trees with no wild EAB larvae and with healthy crowns, suggesting that these trees were able to mount effective defensive responses to the beetles. Indeed, when sampling wild populations in sites with mature EAB infestations tree resistance was a negligible source of mortality.

In conclusion we found that woodpeckers can have a significant effect on EAB larval mortality, although at relatively newly infested sites it does not appear as though woodpeckers exert much of an effect on EAB population growth. A positive response to EAB from woodpeckers could eventually assist with regulating EAB populations, but woodpeckers typically do not forage on ash trees until the trees are already heavily infested and therefore the benefits to these trees are likely to be limited. Furthermore it is possible that woodpecker predation on EAB larvae at the sites of introduced parasitoid releases could adversely affect the establishment of the parasitoids. Because woodpeckers appear to respond strongly to EAB density, it would be interesting for future work to create EAB larval cohorts with a range of densities to explore the functional response of these predators.
